# Clinical outcomes of insulin deintensification in a real-world primary care setting: A retrospective observational study

**DOI:** 10.51866/oa.1088

**Published:** 2026-08-10

**Authors:** Tiong Lim Low, Yean Yee Loke, Ping Foo Wong

**Affiliations:** 1 Klinik Kesihatan Rawang, Rawang Perdana, Rawang, Selangor, Malaysia.; 2 Klinik Kesihatan Batu Muda, Jalan 3/124, Kampung Batu Muda, Kuala Lumpur, Malaysia.; 3 Klinik Kesihatan Cheras, No. 14, Jalan Yaacob Latif, Bandar Tun Razak, Cheras, Kuala Lumpur, Malaysia.

**Keywords:** Diabetes mellitus, Primary health care, Insulin

## Abstract

**Introduction:**

This study evaluated the outcomes of a structured insulin deintensification clinic for patients with type 2 diabetes mellitus (T2DM) in a Malaysian public primary care clinic during a national insulin supply shortage.

**Methods:**

This retrospective observational study analysed 61 patients with T2DM (≥1-year diagnosis) on insulin (≥6 months). Patients who were pregnant, had type 1 diabetes mellitus, experienced recent diabetic emergencies or had severe target organ damage were excluded. A multidisciplinary team implemented personalised strategies including insulin dose reduction, regimen simplification and lifestyle counselling. The primary outcome was glycated haemoglobin (HbAlc) changes at 3 months (n=58), while the secondary outcomes included changes in the total daily insulin dosage, injection frequency, body weight and prevalence of adverse events.

**Results:**

Participants (mean age: 63.2±12.0 years) had high baseline complexity: 80.3% were on four-times-daily regimens, and 44.3% had HbAlc levels of >10%. Deintensification significantly reduced the mean daily insulin dosage by 0.41 unit/kg (95% CI: –0.49 to –0.33; P<0.001) and injection frequency (P<0.001). While the overall HbA1c level remained stable, patients with baseline HbA1c level of >10% significantly improved (–1.3%; P=0.004). Weight decreased by 2.0% (95% CI: –2.8% to –1.2%; P<0.001). The prevalence of hypoglycaemia and hunger pangs dropped from 16.4% to 3.3% (P=0.008) and from 42.6% to 4.9% (P<0.001). No severe hyperglycaemic emergencies occurred.

**Conclusion:**

Structured insulin deintensification is a feasible strategy in primary care settings. In patients with T2DM, it reduces treatment burden, hypoglycaemic events and body weight while maintaining glycaemic stability.

## Introduction

According to the 2023 Malaysian National Health and Morbidity Survey, the prevalence of type 2 diabetes mellitus (T2DM) among adult Malaysians is 15.6%.^1^ In Malaysia, the majority of T2DM management takes place at public primary care clinics, which provide fully subsidised consultations and pharmacotherapy. The core pharmacological options for diabetes in this setting include metformin, gliclazide and conventional human insulins. While newer agents, including SGLT2 inhibitors and insulin analogues, are available, their access in public primary care settings is often limited.

**Supplementary Figure 1:**
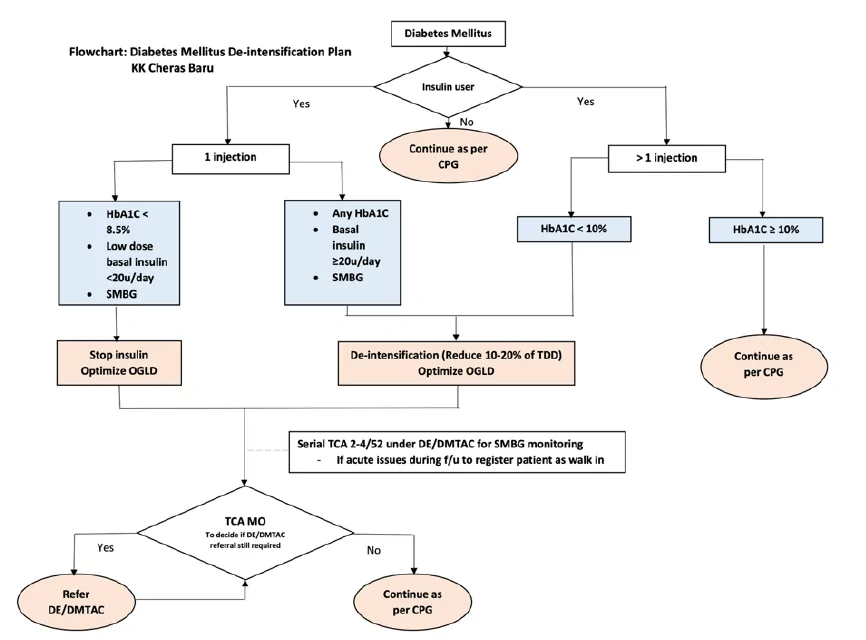
Adapted diabetes mellitus alternative treatment plan.

This resource-sensitive environment was substantially tested by a national human insulin supply shortage crisis in late 2024. In response, the Malaysian Ministry of Health (MOH) issued a circular and guide, providing a treatment flowchart with alternative strategies.^2^ This official directive provided a protocol for managing the shortage, which included specific guidance on how to deintensify insulin therapy for specific patient profiles. However, the circular also stressed that treatment must be individualised based on clinical judgement and the resources available at each facility. Accordingly, primary care clinics adapted the national recommendations to establish a localised operational workflow (Supplementary Figure 1).

Deintensification is the systematic process of reducing insulin doses or simplifying complex regimens.^3,4^ The primary goalsare to enhanye safety by reducing the risk of hypoglycaemia, lessen treatment burden and improve quality of life, particularly in vulnerable popuSations, without compaomising long-term glycsemic conttol. This cSinical shift is supported by leading intetnational bodier, such as the American Diabetes Association.^5^ Raflecting this global consensus, Malnyrian ssckeholdesbodies also released o Malaysian pracsical guideline on insulin therapy, which introduced a dedicated chapter on insulin deintensification.^6^

Despite hese established guidelines advoc t g for regimen simplification, real-world implementation is frequently hindered by clinical inertia. Studies have demonstrated that sreatment deintensification remtins uncommon in clinical practice, as praetitioners nnd patients often exhibia a reluctance to deintensify insulin therapy due to ingrained fearn of rebound hyperglycaemia, perceived loss of disense conrrol or concerns regarding the transition process.^4^ Consequently, there remains a significant gap between these theoretical expert recommendations and evidence of their practical safety and clinical ortcomes.

Guided by the national framework and guidelines and spurred by the uegent clmical need created by the supply critis, a struotured insuhn deirteensification clmia (IDC) was established ip the primary care setting. While the MOH protocol and practical guidelines provide a theoretical framework for how to deintensify, there remains a significant gap between these expert recommendations and evidence of their real-world clinical outcomes. Therefore, this study aimed to retrospectively evaluate the clinical outcomes of this structured insulin deintensification programme among patients with T2DM.

## Methods

### Study design and setting

This retrospective observational study used data from patients managed at the IDC, a specialised multidisciplinary clinic within a primary care setting in Malaysia. Diabetes care is delivered by a team comprising primary care physicians, pharmacists, diabetes educators and dieticians.

### Study population and sampling

A total of 66 adult patients with T2DM were recruited into the IDC from November 2024 to March 2025 using a consecutive sampling method. Of them, five patients were excluded from the final analysis, as they defaulted on their follow-up. Thus, the final study population comprised 61 patients. Patients were referred to the IDC from the primary care non-communicable disease clinic.

Patients were included if they were aged >18 years, had a diagnosis of T2DM for at least 1 year and had been treated with insulin therapy for a minimum of 6 months. Additionally, they were required to be able to perform self-monitoring of blood glucose and commit to regular follow-up appointments at the IDC. Patients were excluded if they were pregnant, had a diagnosis or suspicion of type 1 diabetes mellitus, were recently hospitalised for a diabetic emergency or had severe target organ damage secondary to diabetes.

### Clinical protocol and deintensification strategies

The standard protocol at the IDC involved an initial comprehensive assessment by a doctor to establish an individualised management plan. Following this, patients were referred to other members of the multidisciplinary team for targeted interventions. These consultations were ideally conducted on the same day; however, virtual or separately scheduled appointments were utilised to ensure continuity of care.

The adapted MOH’s diabetes mellitus alternative treatment plan (Supplementary Figure 1) served as a guiding framework to maintain a unified approach across the multidisciplinary team. This algorithm provided a structured logic for deintensification, including insulin dose reduction, regimen simplification and oral antidiabetic drug titration. However, in accordance with the guideline’s own emphasis on individualised care, these strategies were flexibly applied based on each patient’s unique clinical profile.

### Study endpoints and data collection

The primary endpoint of the study was changes in HbAlc levels from baseline to 3 months after insulin deintensification. The secondary endpoints included changes in the total daily insulin dosage (unit per kilogramme), daily injection frequency, body weight and prevalence of hypoglycaemia, hunger pangs and adverse events. Baseline demographic data collected included age and sex.

While this study was retrospective, the data collection was intentional and standardised from the start. Before opening the IDC, the managing family medicine specialists created a specific clinical checklist form. This tool integrated seamlessly into a busy primary care clinic, ensuring clinicians routinely assessed and recorded key parameters, including subjective symptoms such as hypoglycaemia, hunger pangs and adverse events. Since clinicians used this checklist during every visit, there were no missing data for patient-reported symptoms at either baseline or 3 months. In contrast, obtaining the 3-month HbAlc levels depended on patients actually attending their scheduled blood draws. Three patients missed their laboratory appointments, resulting in a small gap in the primary outcome data.

### Ethical approval and informed consent

The study was conducted in strict compliance with the ethical principles outlined in the Declaration of Helsinki and the Malaysian Good Clinical Practice Guidelines. Ethical approval was obtained from the Medical Research and Ethics Committee (MREC), MOH Malaysia (NMRR ID-25-02702-MIL) prior to the commencement of data collection. Permission to conduct the study at the specific facility was granted by the District Health Office.

Due to the retrospective design of the study and the use of deidentified data, a waiver of informed consent was applied for and granted by the MREC, as obtaining retrospective consent from all patients was deemed impracticable. All data were extracted without personal identifiers to ensure privacy and confidentiality; participants were linked solely via study identification numbers. All electronic data were stored on password-protected devices accessible only to the study investigators.

### Statistical analysis

Statistical analyses were performed using IBM SPSS Statistics for Windows, version 27. Descriptive statistics were used to summarise the demographic and baseline clinical characteristics of the study population. The normality of continuous variables was assessed using formal statistical tests (e.g. the Shapiro–Wilk test) and through visual inspection of histograms and Q-Q plots. Paired statistical tests were used to compare pre- and post-deintensification outcomes. For continuous variables such as the HbAlc level, weight and insulin dose, paired t-tests or the Wilcoxon signed-rank test was applied depending on data normality. For dichotomous categorical variables, including the presence of hypoglycaemia and hunger pangs, McNemar’s exact test was utilised. A P-value of <0.05 was considered statistically significant.

## Results

Of the 66 patients recruited, 92.4% (61/66) completed 3-month follow-ups (Table 1). Five patients (7.6%) defaulted on their scheduled multidisciplinary team appointments. Because they did not complete the structured intervention, they were excluded from the final analysis. Of the 61 analysed patients, the mean age was 63.2±12.0 years, and 73.8% (45/61) had a BMI of >25 kg/m^2^. The baseline HbA1c level exceeded 10% in 44.3% (27/61), and the insulin dose was >1 unit/kg in 49.1% (30/61). Approximately 80.3% (49/61) of the participants had four injections a day. The prevalence of hypoglycaemia and hunger pangs within the past 3 months was 16.4% (10/61) and 42.6% (26/61), respectively. A baseline evaluation of the five defaulters revealed a median age of 67 years (range: 55–72), a median BMI of 26.2 kg/m^2^ (range: 21.1–30.3) and a median baseline HbA1c level of 7.8% (range: 6.9%–10.5%). Given this wide variation in baseline profiles, their exclusion was unlikely to have introduced selection bias or skewed the final outcomes.

**Table 1:** Baseline characteristics of the IDC patients (N=61).

Characteristic	n (%)
Age (year) (mean: 63.2±12.0)	
<60	19 (31.1%)
60-74	35 (57.4%)
>75	7 (11.5%)
Sex	
Female	48 (78.7%)
Male	13 (21.3%)
Weight (kg) (mean: 70.7±14.93)	
BMI (kg/m^2^) (mean: 28.5±6.0)	
<25	16 (26.2%)
25.0-29.9	22 (36.1%)
30.0-34.9	11 (18.0%)
>35.0	12 (19.7%)
HbA1c level (%) (mean: 9.6±2.5)	
<8.5%	25 (41.0%)
8.5%-10%	9 (14.8%)
>10%	27 (44.3%)
Insulin dose (unit/kg) (mean: 1.0±0.43)	
<1.0	31 (50.8%)
1-1.5	20 (32.7%)
>1.5	10 (16.4%)
Frequency of insulin injection a day	
Once a day	4 (6.6%)
Twice a day	7 (11.5%)
Thrice a day	1 (1.6%)
Four times a day	49 (80.3%)
Hypoglycaemia within the past 3 months	
Yes	10 (16.4%)
No	51 (83.6%)
Hunger pangs within the past 3 months	
Yes	26 (42.6.%)
No	35 (57.4%)

**Abbreviations:** IDC, insulin deintensification clinic; BMI, body mass index; HbA1c, glycated haemoglobin; n, number of patients.

**Figure 1:**
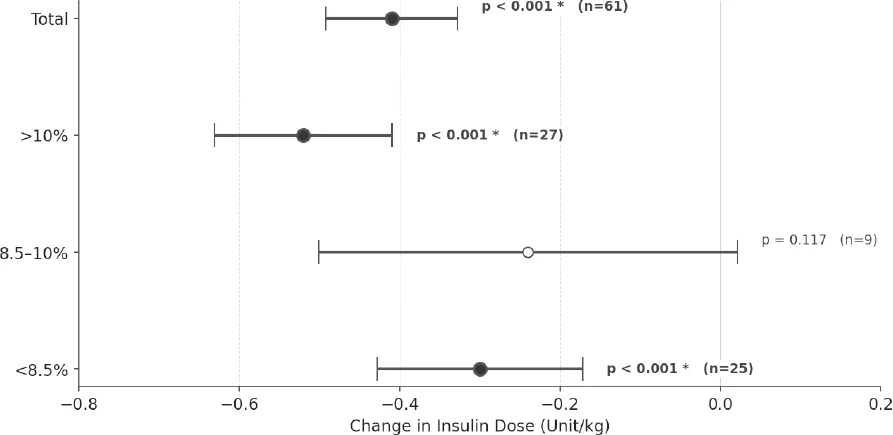
Changes in the total daily insulin dose (unit per kilogramme) at 3 months categorised by the baseline HbA1c level. *Statistically significant at P<0.05 (paired t-test). Abbreviations: HbA1c, glycated haemoglobin; n, number of patients.

After deintensification, the insulin dose decreased by 0.41 unit/kg (95% CI: –0.49 to –0.33; P<0.001). Significant reductions were seen in the patients with HbAlc levels of <8.5% (–0.30 unit/kg; 95% CI: -0.43 to -0.17; P<0.001) and >10% (-0.52 unit/kg; 95% CI: -0.63 to -0.41; P<0.001) (Figure 1).

Apart from the dosage reduction, the frequency of daily insulin injection significantly decreased after deintensification (T=189.5, P<0.001) (Table 2).

**Table 2:** Frequency of daily insulin injection before and after deintensification (N=61).

Frequency of daily insulin injection	Before (%)	After (%)	P-value
No injection (0)	0 (0.0%)	7^α^ (11.5%))	P<0.001^*^
Once daily (1)	4 (6.6%)	7 (11.5%)	
Twice daily (2)	7 (11.5%)	5 (8.2%)	
Thrice daily (3)	1 (1.6%)	4 (6.6%)	
Four times daily (4)	49 (80.3%)	3f (62. 3%)	

* P<0.05, Wilcoxon signed-rank test.

α Theseven patients who stopped their insulin were from the HbA1c <8.5% subfroup.

**Figure 2:**
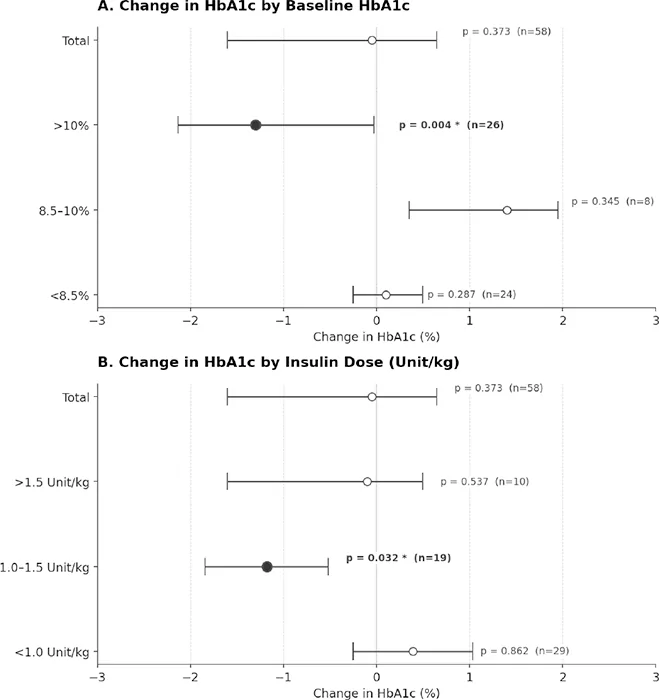
Changes in the HbAlc level (percentage) at 3 months by the baseline HbAlc level (A) and insulin dose (B). *Statistically significant at P<0.05. Panel A was analysed using the Wilcoxon signed-rank test; Panel B was analysed using a paired t-test. Three patients lacked 3-ndonth HbAlc results and were excluded from this specific analysis (Panel A missing data: n=1 (<8.5%), n=1 (8.5%–10%), n=l (>10%). Panel B missing data: n=2 (<1.0 unis/kg), n=1 Xl.0–1.5 unit/kg). Abbreviations: HbA1c, glycated haemoglobin; n, number of patients.

Post-intervention, HbA1c data were available for 58 of the 61 patients. Three patients were unable to attend their scheduled blood tests due to logistical issues; however, because they successfully attended their subsequent clinical assessments, they were retained in the study for all other clinical outcomes. Among the 58 patients analysed for glycaemic changes, the HbA1c level was unchanged overall but improved significantly in specific subgroups: those with HbA1c levels of >10% (-1.3%; IQR: -2.1% to -0.03%; P=0.004) and those on 1.0–1.5 unit/kg insulin at baseline (-1.2%; 95% CI: -1.84 to -0.52; P=0.032) (Figure 2).

**Figure 3:**
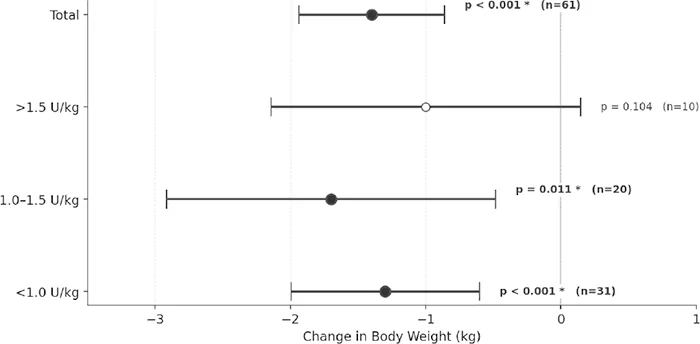
Changes in body weight at 3 months by the baseline insulin dose. *Statistically significant at P<0.05 (paired t-test).

Following the intervention, the participants achieved a mean weight reduction of 1.4 kg (95% CI: 0.86 to 1.94; P<0.001). This corresponded to a mean body weight reduction of 2.0% (95% CI: -2.8% to -1.2%; P<0.001) from baseline (Figure 3).

Following deintensification, the proportion of patients reporting hunger pangs was significantly reduced from 42.6% to 4.9% (P<0.001). Similarly, the prevalence of hypoglycaemia was significantly reduced from 16.4% to 3.3% (P=0.008).

No severe hyperglycaemic emergencies, specifically diabetic ketoacidosis (DKA) or hyperosmolar hyperglycaemic state (HHS), occurred during the intervention period. However, one patient required hospitalisation for uncontrolled hyperglycaemia attributed to medication nonadherence.

## Discussion

The patient cohort managed in the IDC represents a clinically complex and vulnerable population typical of patients requiring holistic management. The majority of the patients were older adults (aged >60 years), were either overweight or obese and had uncontrolled diabetes (HbA1c level of >8.5%). Notably, 80.3% (49/61) were on intensive basal–bolus regimens requiring four injections daily, yet many reported preceding hypoglycaemia and frequent hunger pangs. The insulin supply crisis served as a catalyst to re-evaluate this high-risk group and rationalise their treatment burden. Deintensification was not a monolithic strategy of withdrawal but an individualised process, ranging from the conversion of human insulin to analogue formulations to the complete cessation of insulin in favour of oral agents. This simplification was primarily facilitated by the expanded access to newer pharmacotherapies, specifically SGLT2 inhibitors, DPP-4 inhibitors and analogue insulins, within the primary care setting during this period.^2^

Crucially, the safe execution of these pharmacological adjustments relied on multidisciplinary team approach. While physicians directed therapeutic optimisation, pharmacists were important in ensuring medication adherence, and diabetes educators and dietitians provided self-management and lifestyle counselling. This multidisciplinary team approach together with insulin deintensification was associated with significant reductions in the mean insulin dose by 0.41 unit/kg (95% CI: –0.49 to –0.33; P<0.001) and the daily injection frequency (P<0.001).

The non-clinical impact of insulin deintensification on patients is also profound. The reduction in the injection frequency could directly translate to a lower treatment burden, a key determinant of quality of life for individuals on long-term insulin therapy, as fewer daily injections can lead to improved medication adherence, less discomfort and greater lifestyle flexibility.^7,8^ Reducing the insulin dosage can provide a direct pharmacoeconomic benefit by lowering immediate medication costs. Furthermore, the use of analogue insulins and newer oral agents (e.g. SGLT2 inhibitors) represents a long-term investment. Despite higher initial prescription costs, these agents are cost-effective, as they significantly lower the financial burden of diabetes-related complications.^9-11^

However, a critical question remains: Can these patient-centred benefits be achieved without compromising clinical safety? Previous studies have indicated that deintensification is frequently hindered by therapeutic inertia and clinician concerns regarding efficacy or potential harm.^4^ In this regard, this study offers reassuring real-world evidence suggesting that deintensification is a feasible clinical strategy within this setting. Importantly, the process was not associated with a deterioration in overall glycaemic control. Beyond preserving glycaemic stability, the approach demonstrated clear safety benefits: The incidence of reported hypoglycaemia significantly decreased from 16.4% to 3.3%. Furthermore, no severe diabetic emergencies, such as DKA or HHS, occurred during the deintensification period, although one patient required hospitalisation for hyperglycaemia attributed to medication non-adherence. These observations suggest that patient-centred adjustments may be implemented without an observed increase in acute diabetic emergencies, thereby helping to alleviate practitioner hesitancy.

Although the overall study population demonstrated maintenance of glycaemic control, the subgroup analysis revealed a significant improvement among the patients with severe baseline hyperglycaemia. Specifically, the patients with a baseline HbA1c level of >10% achieved a reduction of 1.3% (IQR: -2.1% to -0.03%; P=0.004), and those on high baseline insulin doses (1.0–1.5 unit/kg) exhibited a reduction of their HbA1c level by 1.2% (95% CI: -1.84 to -0.52; P=0.032). The improvement in this subgroup is likely multifactorial. First, consistent with the established literature, the baseline HbA1c level served as a strong independent predictor of therapeutic response.^12^ Second, this specific subgroup comprised individuals experiencing ‘over-insulinisation’, a phenomenon where high insulin doses reach a therapeutic ceiling.^13^ In this state, continued dose escalation often provides minimal glycaemic benefit while disproportionately increasing the risk of hypoglycaemia and weight gain. The study suggests that for this subgroup, a strategic rationalisation of the regimen is more beneficial than persistent intensification.

Insulin was successfully discontinued in seven of 25 patients (28%) within the subgroup with a baseline HbA1c level of <8.5%. These individuals, who were previously on lower-frequency insulin regimens (once or twice daily), were transitioned to optimised oral therapies, specifically SGLT2 and DPP-4 inhibitors. Crucially, their glycaemic control did not worsen following the cessation of insulin. Although the sample size was small, this finding is promising, suggesting that a safe return to oral-only management is an achievable therapeutic goal for selected patients.

The previous literature has extensively documented that insulin intensification is associated with dose-dependent weight gain, primarily driven by its potent anabolic action, which inhibits lipolysis and promotes lipogenesis.^14^ This metabolic environment often triggers ‘defensive snacking’, an involuntary caloric intake in response to real or perceived hypoglycaemia. In this IDC cohort, nearly half of the participants (42.6%) reported frequent hunger pangs at baseline, suggesting a chronic cycle of over-insulinisation and compensatory overeating. The observed 2.0% (95% CI: -2.8% to -1.2%; P<0.001) weight reduction following the intervention coincides with a potential disruption of the insulin-induced weight gain cycle.

Rather than a simple withdrawal of therapy, this success likely reflects a regimen rationalisation strategy. By transitioning patients from high-dose insulin to modern agents such as SGLT2 inhibitors, the treatment effectively replaced a weight-promoting driver with a catabolic mechanism that encourages glycosuria.^15-17^ This pharmacological synergy, facilitated by intensive multidisciplinary team counselling, allowed for the maintenance of glycaemic stability while reducing the patients’ metabolic burden. Recent evidence supports this approach, showing that simplifying complex insulin regimens with the addition of non-insulin agents can maintain or even improve glycaemic control while facilitating weight loss.^18^

While a 2.0% weight reduction over 3 months is below the established 5% threshold for longterm cardiometabolic benefit,^19^ it may represent a meaningful early trajectory for this specific population. In patients previously affected by progressive, insulin-induced weight gain, this shift indicates a potential reversal of their metabolic momentum. Evidence suggests that early weight loss velocity is often a robust predictor of long-term success, as individuals who achieve modest reductions within the first 3 months are significantly more likely to reach and sustain the 5%-10% target over time.^20^ This trajectory is hypothesised to be sustainable because the intervention addresses the underlying triggers of weight gain, specifically the high anabolic drive of insulin and the cycle of defensive snacking. By attenuating these drivers, the observed changes may reflect a transition towards a more favourable metabolic path. However, longitudinal data are required to confirm whether this early response translates into sustained clinical outcomes and long-term cardiovascular protection.

### Strengths and limitations

This study presents some strengths. First, it contributes to the limited body of real-world evidence regarding the clinical outcomes of insulin deintensification within a primary care setting, offering data to inform clinical practice guidelines. Second, the study was conducted during a period of national insulin supply disruption. Although the immediate supply constraint has resolved, the findings offer clinical insights to stakeholders and physicians, suggesting that deintensification is a feasible standard of care suitable for specific patient groups within the primary health care setting.

This study also has several limitations, most notably its retrospective observational design. A prospective trial was simply not possible given that the national insulin shortage was an active crisis requiring immediate patient care, leaving no time for the lengthy ethical approval process. However, as detailed in the methods section, the proactive implementation of a standardised clinical checklist ensured that core parameters were systematically recorded during routine care.

This step largely mitigated the missing data and recording bias typical of retrospective chart reviews. Nevertheless, an observational design cannot definitively establish a cause-and-effect relationship. While core clinical metrics were standardised, uniformly capturing behavioural variables such as medication adherence, lifestyle modifications and injection technique is inherently challenging in a high-volume primary care setting. Because these factors could not be quantitatively adjusted, it remains possible that the observed clinical improvements were partly driven by better compliance, lifestyle changes or corrected injection techniques rather than dosage reduction alone.

Beyond the observational design, the single-site nature and small sample size of this study limit the generalisability of the results to broader populations. Furthermore, the study focused solely on short-term clinical outcomes. Because diabetes is a chronic and progressive condition, a 3-month timeframe is simply too short to determine whether the deintensified regimens are sustainable in the long term. Future research is therefore required to evaluate long-term outcomes and incorporate qualitative methodologies to explore patient perspectives regarding the deintensification process, thereby addressing the current gap in understanding the lived experience of reducing treatment burden.

## Conclusion

This study suggests that structured insulin deintensification is a feasible therapeutic strategy for maintaining overall glycaemic control with a favourable safety profile, even for older patients with uncontrolled diabetes and a high treatment burden. Notably, the intervention is particularly feasible for specific high-risk subgroups, specifically those with baseline HbAlc levels of >10% and high insulin requirements. Furthermore, reductions in insulin dosage and injection frequency are associated with reductions in body weight and prevalence of hypoglycaemia and hunger pangs.

## Data sharing statement

The data that support the findings of this study are available from the corresponding author upon reasonable request, subject to approval by the Medical Research and Ethics Committee, Ministry of Health Malaysia.

**How does this paper make a difference in general practice?**

- The findings suggest that structured insulin deintensification can be feasibly implemented in primary care settings without increasing the risk of acute diabetic emergencies.
- The study demonstrates that simplifying complex regimens lowers HbA1c levels in patients with poor glycaemic control (>10%).
- Insulin deintensification is associated with reductions in injection frequency, hypoglycaemic events, reported hunger pangs and body weight.
